# Effects of different doses of glucocorticoids on postoperative atrial fibrillation: a meta-analysis

**DOI:** 10.1186/s12872-022-03001-0

**Published:** 2023-01-12

**Authors:** Zhongzheng Zhou, Yi Long, Xin He, Yong Li

**Affiliations:** Department of Cardiology, Chongqing Traditional Chinese Medicine Hospital, Chongqing, 400025 China

**Keywords:** Glucocorticoids, Postoperative atrial fibrillation, Inflammatory, Cardiac surgery, Meta- analysis

## Abstract

**Background:**

Postoperative atrial fibrillation (POAF) is the most common arrhythmia after cardiac surgery, and its occurrence is closely related to inflammation. This paper intends to apply meta-analysis to investigate the effect of glucocorticoids on POAF.

**Methods:**

PubMed, Embase, Web of Science, and Cochrane Library were searched using the internationally recognized systematic evaluation and retrieval strategy. Two review authors independently selected relevant studies and extracted data based on the Cochrane handbook for systematic reviews of interventions approach. Stata 17 was used for data analysis. In the subgroup analysis, we grouped the participant data according to differences in glucocorticoids dose and type of surgery. At the same time, we also conducted a meta-analysis on the possible infection and gastrointestinal injury caused by glucocorticoids use.

**Results:**

27 studies and 14,442 patients were finally included. Results from the random-effects model indicated that the incidence of POAF was lower in glucocorticoid group (RR 0.80, 95% CI 0.71–0.92, *P* = 0.001). According to the subgroup analysis result, low doses of glucocorticoids reduced the incidence of POAF (RR 0.81, 95% CI 0.71–0.92, *P* = 0.001). The effect of high doses glucocorticoids on the POAF was not statistically significant (RR 0.81, 95% CI 0.56–1.19, *P* = 0.286). In the coronary artery bypass grafting (CABG) subgroup, the glucocorticoids reduced the incidence of POAF (RR 0.71, 95% CI 0.58–0.87, *P* = 0.001). In the CABG OR Valvular Surgery group, the effect of glucocorticoids on POAF was not statistically significant (RR 0.88, 95% CI 0.75–1.03, *P* = 0.108). 15 studies documented postoperative complications of infection, two studies were excluded from the system because the end point event was 0, and meta-analysis showed no increased risk of infection from glucocorticoid use (RR 0.85, 95% CI 0.68–1.06, *P* = 0.158). Eight studies documented the effects of glucocorticoids on gastrointestinal diseases, and meta-analysis showed no differences between the two groups (RR 1.12, 95% CI 0.83–1.50, *P* = 0.450).

**Conclusion:**

The use of glucocorticoids can reduce the incidence of POAF. The subgroup analysis result showed that low-dose glucocorticoids were more effective than high-dose glucocorticoids in inhibiting POAF. The use of glucocorticoids in CABG alone can better inhibit the occurrence of POAF. The effects of glucocorticoids on infection and gastrointestinal injury were not statistically significant.

*Review registration*: PROSPERO, CRD42022304521.

**Supplementary Information:**

The online version contains supplementary material available at 10.1186/s12872-022-03001-0.

## Introduction

POAF is the most common arrhythmia after CABG and valve surgery, with an overall incidence of 20% to 40% and a higher incidence of approximately 30% to 50% in valve surgery [1]. POAF is closely relevant to stroke, hospital stay time, and the cost of treatment [2, 3]. The pathological mechanism of POAF is very complex, and it is related to many factors. At present, the study about POAF is increasing rapidly, but no clear conclusion has been reached. Relevant research found that the main influencing factors are structure and electrical reconstruction. Furthermore, an increased adrenergic tone and changes in metabolic milieus are also possible mechanisms of POAF [4].

Data suggests that inflammation might lead to the POAF [5]. Trauma, ischemia/reperfusion, and cardiopulmonary bypass during cardiac surgery can induce systemic inflammation; this inflammatory response is closely related to the occurrence of POAF [6–9]. Glucocorticoids can inhibit inflammation and, as a result, may decrease the POAF risk. The effects of glucocorticoids on POAF have been researched widely in many countries. According to previous study result, glucocorticoids reduce postoperative C-reactive protein (CRP) levels, leukocytes, and other inflammatory markers; but the conclusions of these studies are controversial [10, 11]. Thus, we conducted this meta-analysis to investigate the effect of glucocorticoids on POAF.

## Methods

### Literature search strategy and criteria

PubMed, Embase, and Cochrane Library were searched based on internationally recognized systematic evaluation and retrieval strategy. Retrieval of data began from the establishment of the database through December 2021. The search terms were as follows: “Postoperative atrial fibrillation”, “POAF”, “Cardiac surgery”, “CABG”, “Cardiac valve surgery”, “atrial fibrillation”, “AF”, “Glucocorticoids”, “Glucocorticoid”, “Steroid”, “Corticosteroid” and other individual drug names. Articles were also identified by referring to the references of each study to supplement the data.

### Inclusion and exclusion criteria

*Inclusion criteria:* (1) randomized controlled trial (RCT) with glucocorticoids treatment and placebo control; (2) all patients required surgical treatment and had no atrial fibrillation before surgery, where the types of procedures included were CABG, cardiac valve surgery, and CABG combined with valve surgery; (3) published literature.

*Exclusion criteria:* (1) age less than 18 years; (2) cardiovascular risk factors were not considered as primary or secondary endpoints; (3) the full text of the study is not available.

### Quality assessment and data extraction

This meta-analysis was reported per the Preferred reporting items for systematic reviews and Meta-Analyses (PRISMA) guidelines.

**Fig. 1:**
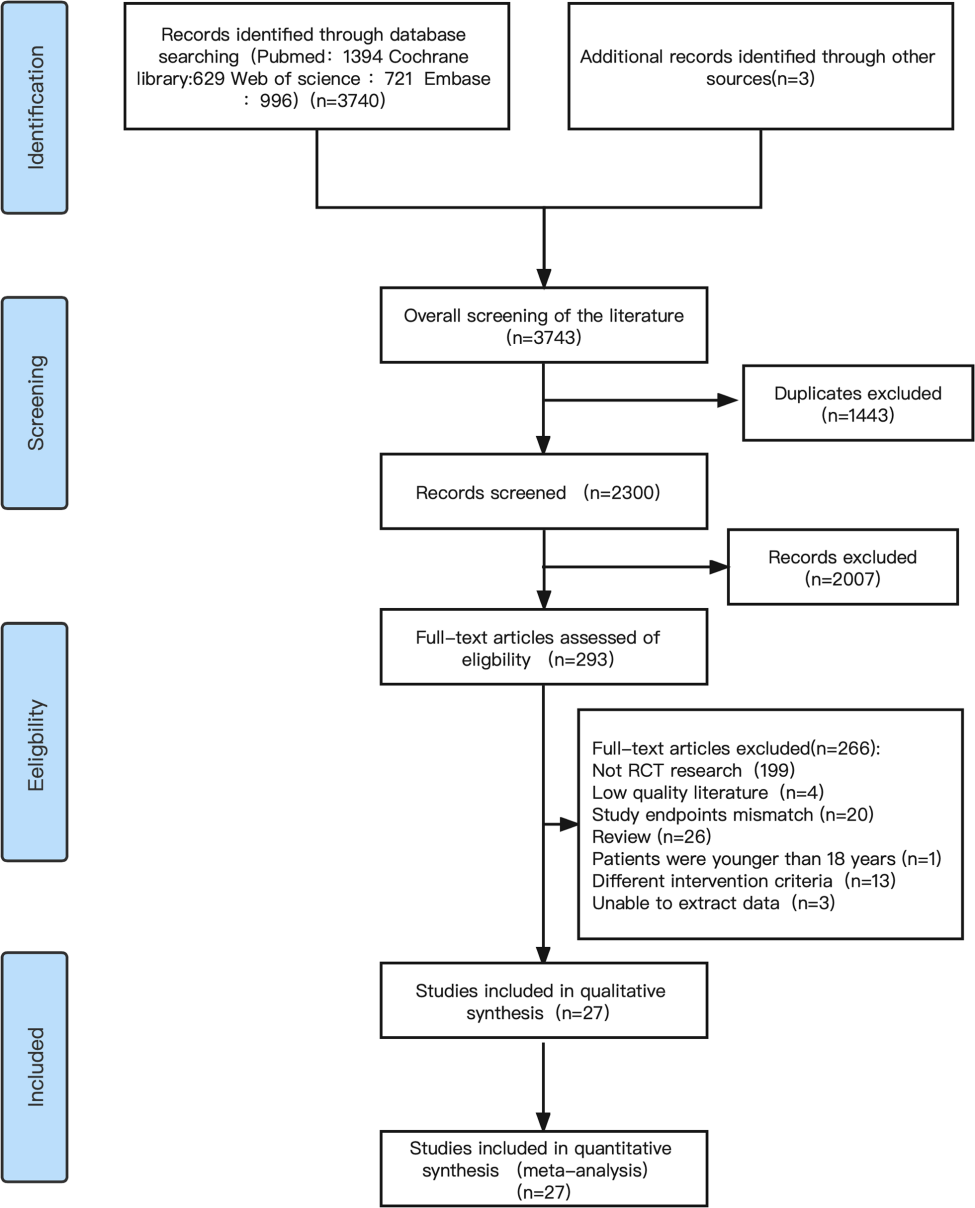
Flowchart of literature retrieval

The data collected in this study were screened and extracted by 2 researchers (Zhongzheng Zhou and Xin He) in strict accordance with the exclusion criteria according to the established retrieval strategy. The Cochrane risk-of-bias assessment tool (RoB 2) was used to evaluate literature bias [12]. Figure 1 shows the flowchart of the literature retrieval (Additional file 1).

### Statistical treatment

In this study, the correlation of glucocorticoids and POAF risk was evaluated by indexes of relative risk (RR) and 95% confidence interval (CI). The RR was used to evaluated glucocorticoids group and placebo group, the glucocorticoids group refers to the use of glucocorticoids before or after surgery, while placebo group refers to the use of an equal dose of saline as placebo. Both Cochran’s Q test and I^2^ index were applied to measure the heterogeneity, where heterogeneity was quantified by I^2^, the range of which is 0 to 100% (judge criteria I^2^ = 0–25%, no; I^2^ = 25–50%, low; I^2^ = 50–75%, moderate; I^2^ = 75–100%, high heterogeneity). In the comprehensive effect study, the random effect model was selected, and subgroup analysis was conducted according to different doses. On this basis, the possible sources of heterogeneity were discussed. The possibility of publication bias was evaluated by Egger test method [13]. The sensitivity is determined by excluding one study at a time, and then the impact of a single study on the overall research results is analyzed. The judgment basis of statistical difference was set as *P* < 0.05. Stata 17 software was used for data analysis.

In addition, two subgroup analyses were conducted, the first subgroup research was conducted based on the dose of glucocorticoids. Patients who were administered dexamethasone doses lower or equal to 100 mg were placed in the low-dose group, and patients who were administered dexamethasone doses higher than 100 mg were classified into high-dose group (where different types of glucocorticoids are converted to equivalent doses of dexamethasone). Previous studies indicated that 100 mg dexamethasone might achieve a balance between efficacy and safety, so we used the 100 mg dose as the standard for subgroup analysis [39]. Second, we divided them into CABG group and CABG OR Valvular surgery group for subgroup analysis according to different surgical types, where the CABG group refers to patients who underwent CABG alone, and the CABG OR Valvular surgery group refers to patients who had valve surgery or CABG combined with valve surgery.

## Results

### Literature search

The literature search process is depicted in Fig. 1. 3743 studies were identified by searching each database according to the search keywords. The titles, abstracts, and full texts of the initially detected literature were read, and 27 studies were included based on above criteria [14–40].

### Study characteristics

**Fig. 2:**
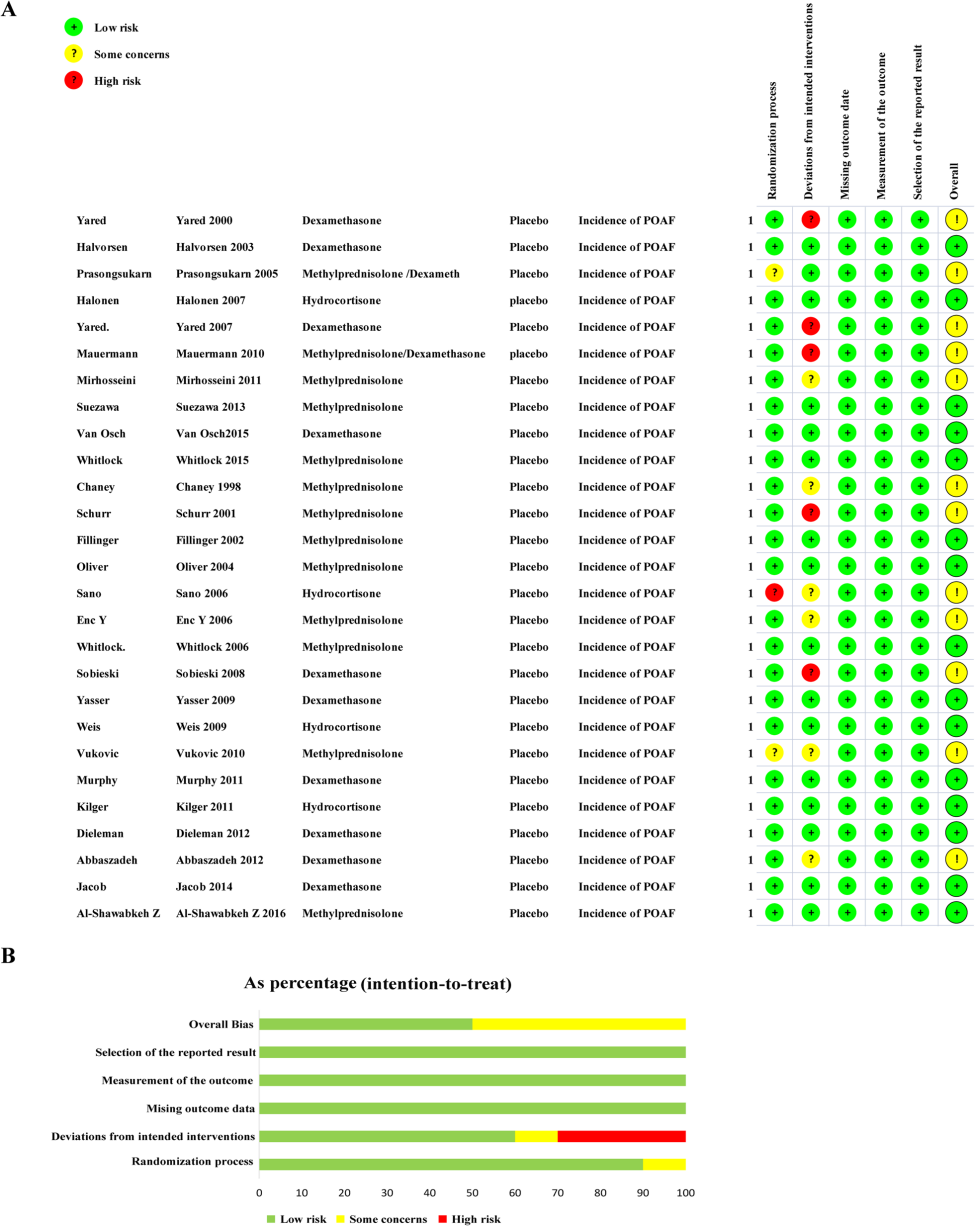
Quality assessment of each included study. **A** Risk bias plot, **B** Summary of risk of bias

Across the included studies, 14,442 patients were enrolled. Table 1 shows the basic feature and quality assessment results of the selected studies. The regions involved in these studies mainly include the EU, Asia and USA Interventions in the trial group included dexamethasone, methylprednisolone, and hydrocortisone. Figure 2 summarizes the results of quality assessment of each study according to the Cochrane risk-of-bias assessment tool (RoB 2).

**Table 1:** Basic information of the studies

Author	Year	Country	Sample size	Age	Type of surgery	Cardiopulmonary bypass	Type and dose of glucocorticoid	Placebo control	Main outcome evaluation time
Chaney	1998	The United States	60	66.5	CABG	Yes	Methylprednisolone 30 mg/kg IV before surgery	Isodose normal saline	Not mention
Yared	2000	The United States	216	63.2	CABG OR Valvular Surgery	Yes	Dexamethasone 0.6 mg/kg IV before surgery	Isodose normal saline	The first 72 h after surgery
Schurr	2001	Swiss	50	62.4	CABG	Yes	Methylprednisolone 10 mg/kg IV before surgery	Isodose normal saline	The first 72 h after surgery
Fillinger	2002	The United States	20	65.2	CABG	Yes	Methylprednisolone 15 mg/kg IV before surgery,0.3 mg every 6 h during the next 24 h	Isodose normal saline	The first 72 h after surgery
Halvorsen	2003	Norway	294	64	CABG	Yes	Dexamethasone 4 mg IV before surgery,4 mg IV after surgery	Isodose normal saline	The first 72 h after surgery
Oliver	2004	The United States	121	61.2	CABG OR Valvular Surgery	Yes	Methylprednisolone 1 g IV before surgery and of dexameth asone 4 mg every 6 h for 1 day after surgery	Isodose normal saline	The first 72 h after surgery
Prasongsukarn	2005	Canada	86	64.4	CABG	Yes	Methylprednisolone 1 g IV before surgery and of dexameth asone 4 mg every 6 h for 1 day after surgery	Isodose normal saline	The first 7 days after surgery
Sano	2006	Japan	60	62.5	CABG	Yes	Hydrocortisone 50 mg/kg IV before surgery	Isodose normal saline	The first 72 h after surgery
Enc Y	2006	Turkey	40	58.3	CABG	Yes	Methylprednisolone 25 mg/kg IV before surgery	Isodose normal saline	The first 5 days after surgery
Whitlock	2006	Canada	51	66.5	CABG	Yes	Methylprednisolone 250 mg IV before surgery	Isodose normal saline	The first 4 days after surgery
Halonen	2007	Finland	241	65	CABG OR Valvular Surgery	Yes	Hydrocortisone 100 mg IV before surgery, 1 dose every 8 h during the next 3 days	Isodose normal saline	The first 84 h after surgery
Yared	2007	The United States	71	71.5	CABG OR Valvular Surgery	Yes	Dexamethasone 0.6 mg/kg IV before surgery	Isodose normal saline	The first 3 days after surgery
Sobieski	2008	The United States	28	63.2	CABG	Yes	Dexamethasone 100 mg IV before surgery	Isodose normal saline	Not mention
Yasser	2009	Egypt	100	67.5	CABG OR Valvular Surgery	Yes	Dexamethasone 1 mg/kg IV before surgery,0.5 mg/kg IV every 8 h	Isodose normal saline	Not mention
Weis	2009	Germany	26	68	CABG	Yes	Hydrocortisone 100 mg IV before surgery, 10 mg/kg per hour after surgery for 24 h	Isodose normal saline	Not mention
Mauermann	2010	The United States	121	62.8	CABG OR Valvular Surgery	Yes	Methylprednisolone 1 g IV before surgery, dexamethasone 4 mg IV every 6 h during the next 24 h	Isodose normal saline	Not mention
Vukovic	2010	Serbia	57	61	CABG	Yes	Methylprednisolone 10 mg/kg IV before surgery	Isodose normal saline	The first 3 days after surgery
Murphy	2011	The United States	98	63.1	CABG	Yes	Dexamethasone 4 mg IV before surgery	Isodose normal saline	The first 3 days after surgery
Mirhosseini	2011	Iran	120	62	CABG	No	Methylprednisolone 5 mg/kg IV before surgery	Isodose normal saline	Not mention
Kilger	2011	Germany	305	68.5	CABG	No	Hydrocortisone 100 mg IV before surgery, 10 mg/kg per hour after surgery for 24 h	Isodose normal saline	Not mention
Dieleman	2012	Netherlands	4482	66.2	CABG OR Valvular Surgery	Yes	Dexamethasone 1 mg/kg IV before surgery	Isodose normal saline	The first 30 days after surgery
Abbaszadeh	2012	Iran	184	60.1	CABG	Yes	Dexamethasone 12 mg IV before surgery	Isodose normal saline	The first 7 days after surgery
Suezawa	2013	Japan	30	68.5	CABG	No	Methylprednisolone 1 g IV before surgery	Isodose normal saline	Not mention
Jacob	2014	Netherlands	62	69.6	CABG OR Valvular Surgery	Yes	Dexamethasone 1 mg/kg IV before surgery	Isodose normal saline	The first 7 days after surgery
Van Osch	2015	Netherlands	1316	66.1	CABG OR Valvular Surgery	Yes	Dexamethasone 1 mg/kg IV before surgery	Isodose normal saline	The first 30 days after surgery
Whitlock	2015	Canada	7507	67.4	CABG OR Valvular Surgery	Yes	Methylprednisolone 500 IV before surgery	Isodose normal saline	The first 4 days after surgery
Al-Shawabkeh Z	2016	Saudi Arabia	340	64.9	CABG	Yes	Methylprednisolone 1 g IV before surgery, dexamethasone 4 mg IV every 8 h during the next 3 day	Isodose normal saline	The first 7 days after surgery

### Correlation between glucocorticoids and POAF

**Fig. 3:**
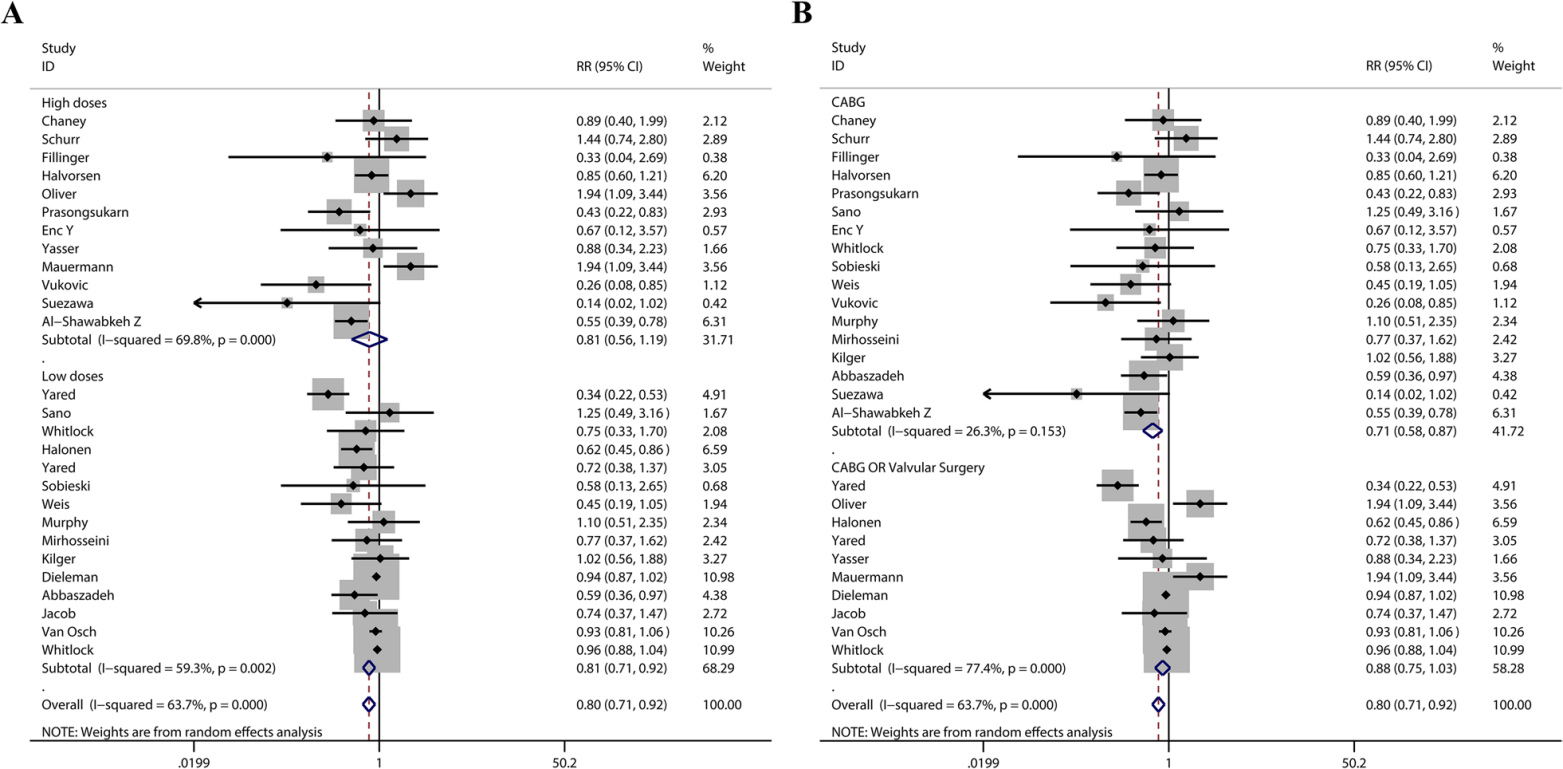
**A** Meta-analysis of the correlation of the first subgroup. **B** Meta-analysis of the correlation of the second subgroup

A total of 27 studies evaluated the influence of glucocorticoids on POAF; in total, there were 7260 cases in glucocorticoids group and 7182 cases in control group. According to the result of Fig. 3, incidence of POAF in glucocorticoid group is lower (RR 0.80, 95% CI 0.71–0.92, *P* = 0.001). In certain range, low doses glucocorticoids can reduce the POAF risk (RR 0.81, 95% CI 0.71–0.92, *P* = 0.001). The risk of POAF was not significantly different between the high-dose glucocorticoids group and the placebo group (RR 0.81, 95% CI 0.56–1.19, *P* = 0.286). In the CABG subgroup, the glucocorticoids reduced the POAF risk (RR 0.71, 95% CI 0.58–0.87, *P* = 0.001). In the CABG OR Valvular Surgery group, the effect of glucocorticoids on POAF was not statistically significant (RR 0.88, 95% CI 0.75–1.03, *P* = 0.108) (see Table 2).

**Table 2:** Subgroup analysis of the of the primary outcome

	Number	RR (95%CI)	*P* value	I^2^	P for I^2^
*Glucocorticoids dose*					
High doses Dexamethasone ≥ 100 mg	12	0.81 (0.56–1.19)	0.286	69.8%	< 0.001
Low doses Dexamethasone < 100 mg	15	0.81 (0.71–0.92)	0.001	59.3%	0.002
*Type of surgery*					
CABG	17	0.71 (0.58–0.87)	0.001	26.3%	0.153
CABG OR Valvular Surgery	10	0.88 (0.75–1.03)	0.108	77.4%	< 0.001

**Fig. 4:**
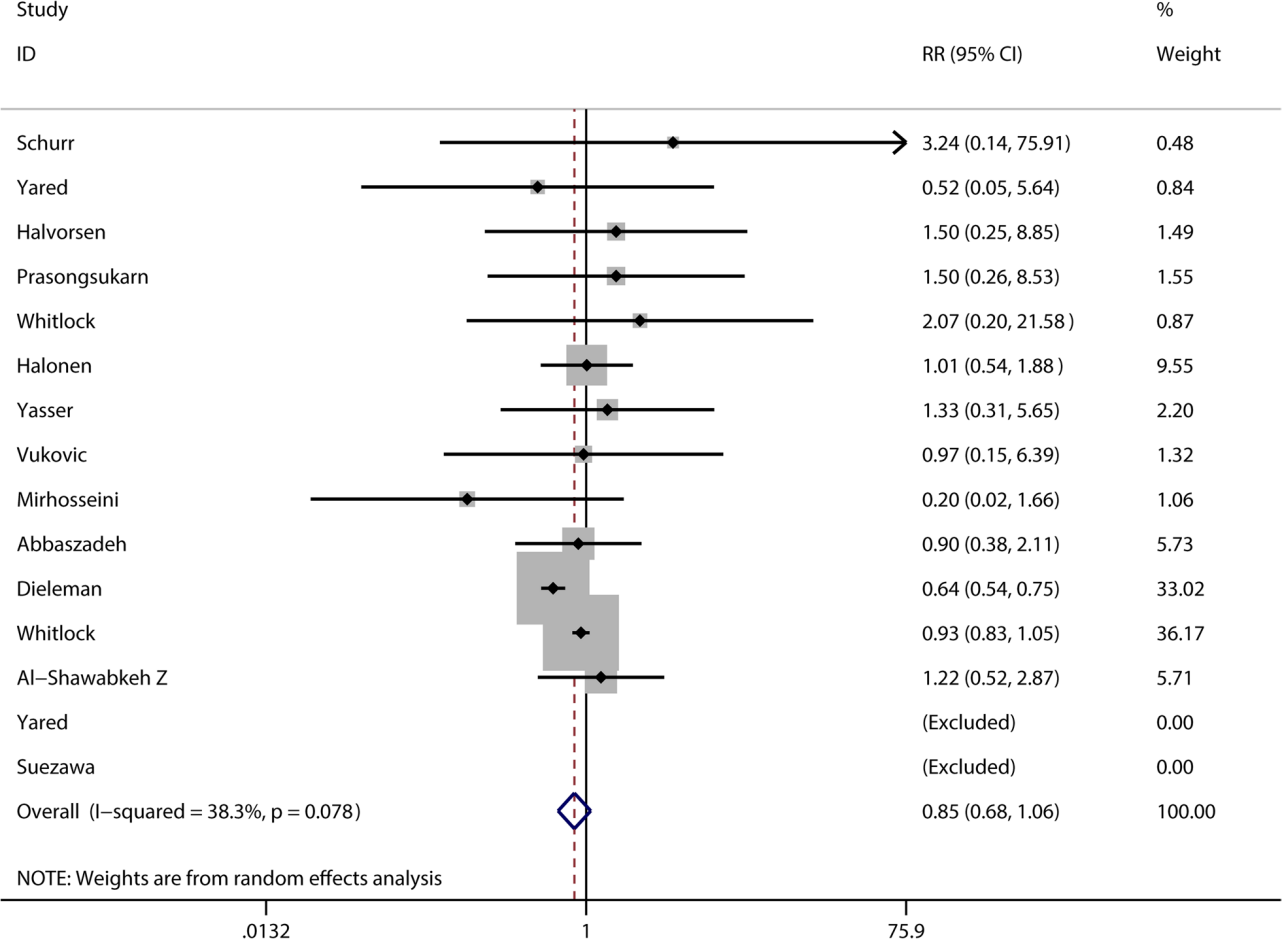
Meta-analysis of the correlation of glucocorticoids and postoperative infection

**Fig. 5:**
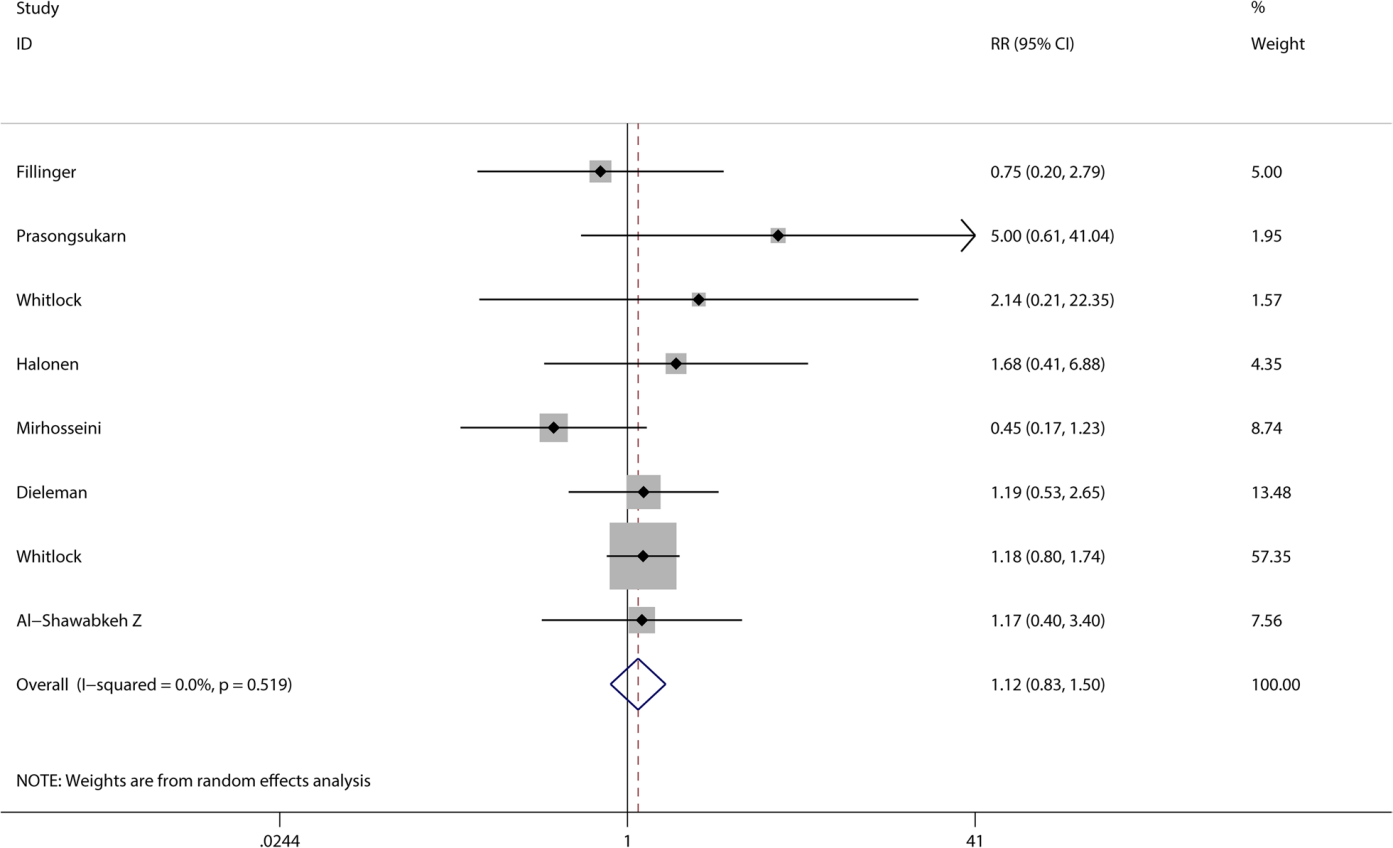
Meta-analysis of correlation of glucocorticoids and gastrointestinal injury

A total of 15 studies documented postoperative complications of infection. However, two studies were excluded from the system because the end point event was 0, and meta-analysis showed no increased risk of infection from glucocorticoid use (RR 0.85, 95% CI 0.68–1.06, *P* = 0.158) (see Fig. 4). And 8 studies documented the effects of glucocorticoids on gastrointestinal diseases, and meta-analysis showed no differences between the glucocorticoid and the control group (RR 1.12, 95% CI 0.83–1.50, *P* = 0.450) (see Fig. 5).

### Publication bias evaluation and sensitivity analysis

**Fig. 6:**
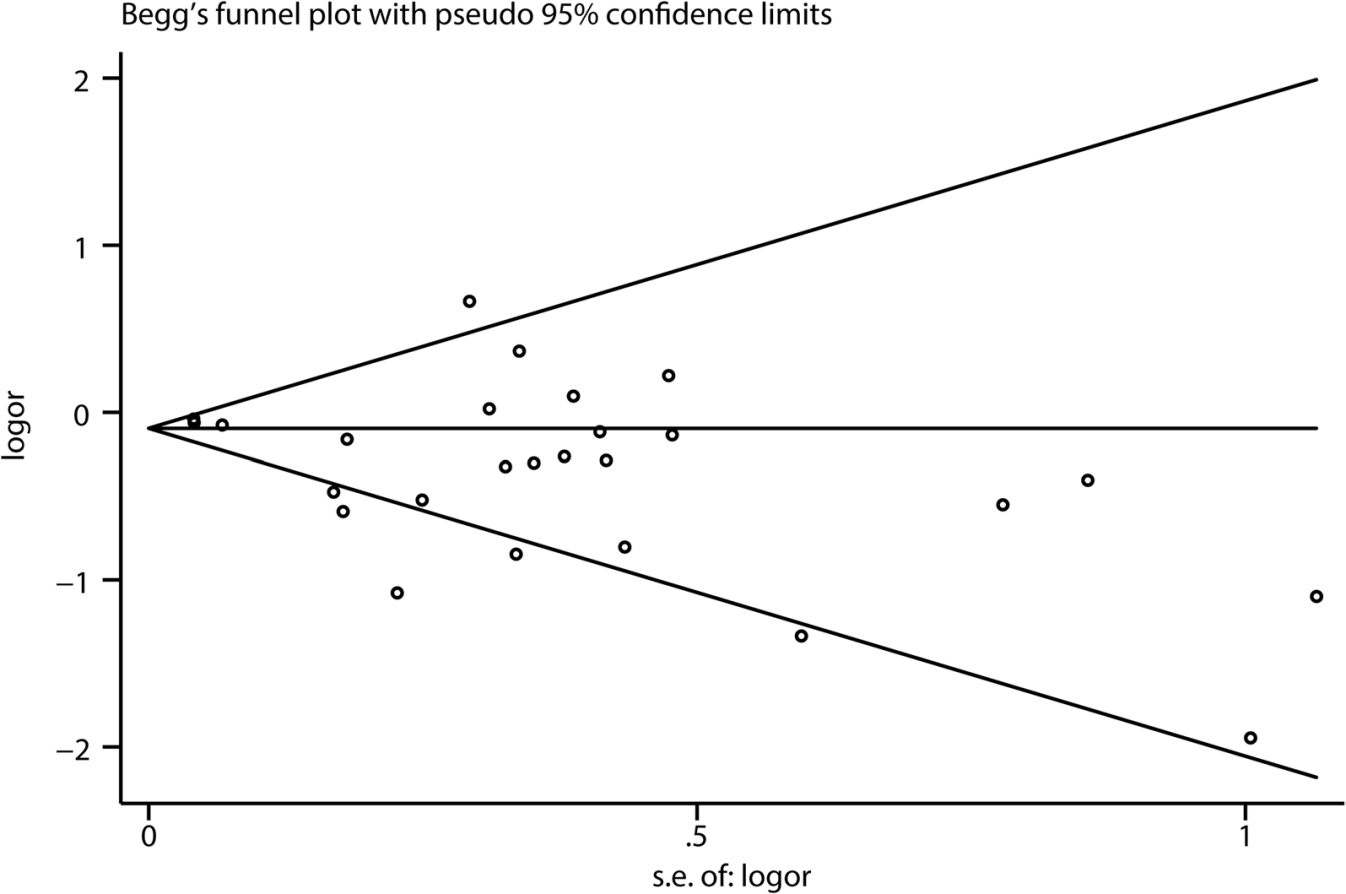
Egger’s publication bias plot of glucocorticoids and POAF

**Fig. 7:**
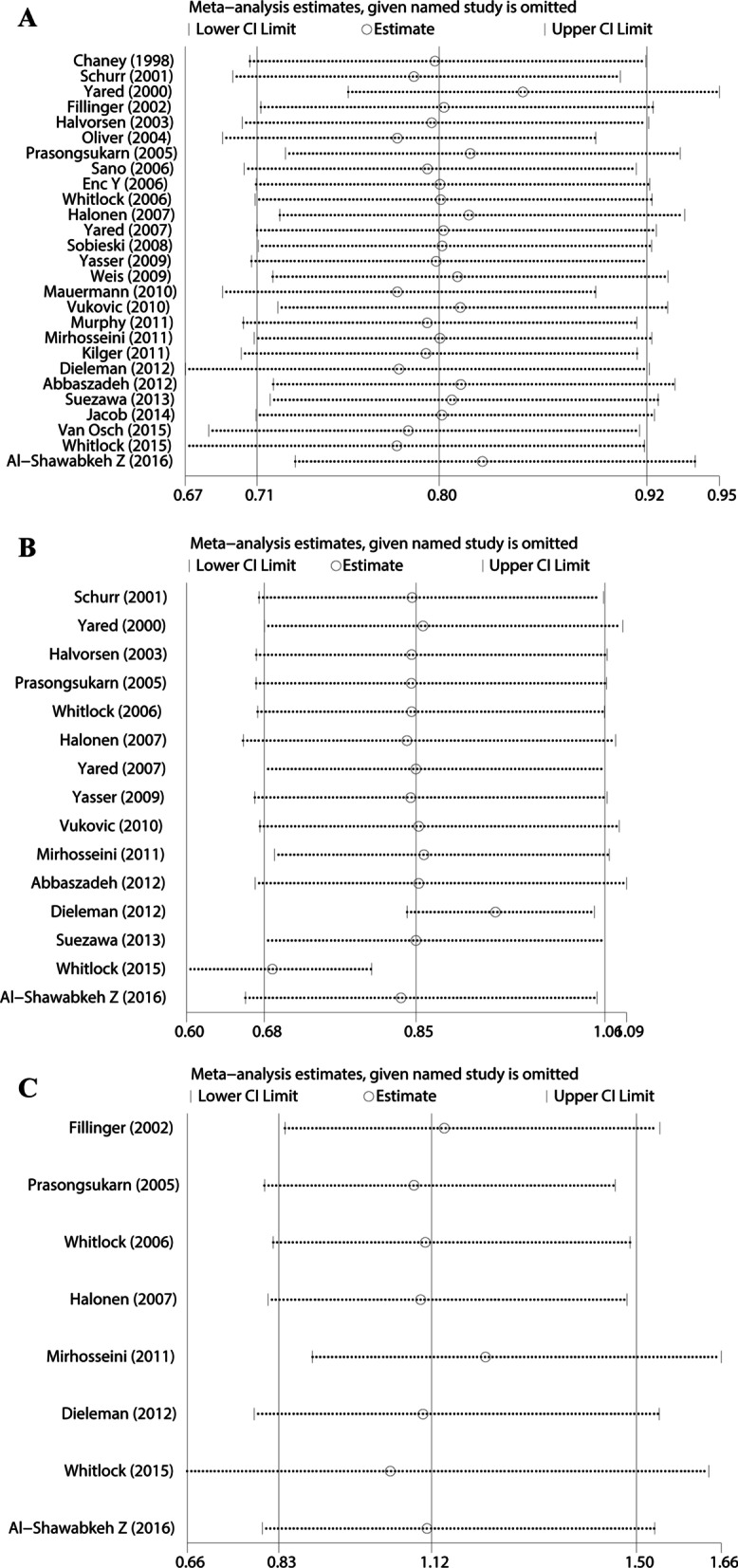
**A** Sensitivity analysis of glucocorticoid to POAF; **B** Sensitivity analysis of glucocorticoid to infection; **C** Sensitivity analysis of glucocorticoid to gastrointestinal injury

In the research process, publication bias were evaluated based on Egger’s test, and the relevant result is shown in Fig. 6. The results of Egger's test were close to the critical value, suggesting that there might be publication bias in the analysis results (*P* = 0.050). Sensitivity analysis was conducted on the three study endpoints. We used the leave-one-out analysis method for sensitivity analysis. The vertical axis represents the study type, and the horizontal axis represents the combined results after excluding a certain study. Sensitivity analysis results showed that excluding any study, the combined results of the remaining studies would not affect the end point outcome, proving that the conclusion was stable (see Fig. 7).

## Discussion

Meta-analysis was used to judge the effect of glucocorticoids use on POAF. The results showed a lower POAF risk in glucocorticoids group, while subgroup analysis showed that low-dose glucocorticoids decreased the incidence of POAF compared to high-dose glucocorticoids. Previous studies on the influence of glucocorticoids on POAF were controversial; some previous studies have yielded positive results, while others have been negative. Past studies included small sample sizes, Van Osch's study was a single-center substudy of the DECS study [38], and the SIRS study [39] was a large controlled trial. Their results suggest that glucocorticoids have no correlation with the incidence of POAF. But the controlled experiment conducted by Al-Shawabkeh in 2016 suggested that glucocorticoids reduced the incidence of that [40]. Therefore, we included patients with all types of surgery for this meta-analysis.

POAF usually occurs during the initial 4 days after cardiac surgery; it is closely related to the type of surgery, with an incidence about (20–30%) in CABG surgery, (30–50%) in valvular surgery and a higher incidence in valvular combined coronary artery bypass surgery (60–80%) [41, 42]. Some school found that the occurrence of POAF is closely related to the prolongation of postoperative hospital stay and the increases in treatment cost [43, 44]. POAF is usually self-limited, and most of cases return to normal on their own within 24 h of onset [45]; although some patients will relapse within 2 days of the first attack [46]. The pathogenesis of POAF is mainly related to local inflammatory reaction [47, 48], adrenergic activation [49], electrolyte disturbances [50], atrial stimulation [51] and prolonged mechanical ventilation [42]. Among them, the inflammatory response is considered to be the most important pathogenesis of POAF [52]. The concentrations of CRP [53], number of white blood cells in POAF patients are higher than those in patients with sinus rhythm [54, 55]; the increase of these inflammatory indicators is closely related to the occurrence of POAF [56, 57]. Glucocorticoids can effectively reduce inflammatory reactions [58], thus reducing the incidence of POAF.

The anti-inflammatory mechanism of glucocorticoids is mainly through inhibiting the activity of immunomodulatory transcription factors such as NF-KB,AP-1, which is dose-dependent. The higher the dose, the stronger the inhibition effect [59]. But in our study, it was found that compared to the high-dose glucocorticoids, low-dose glucocorticoids were more effective in inhibiting the occurrence of POAF. There are a few possible causes for this observation. First, high doses of glucocorticoids cause high expression of calcium regulator mRNA, which can shorten the action potential refractory period and induce arrhythmia. Oakley analyzed the signal pathways related to dysregulated genes, and the results showed that the mechanism of glucocorticoids on POAF might be as follows: improving the expression of voltage-gated L-type calcium channel (LTCC), sarcoplasmic/endoplasmic reticulum calcium adenosine triphosphatase 2 (SERCA2), sodium/calcium exchanger 1 (NCX1) and RyR2 genes, this result has important reference value for the pathological study of POAF [60, 61]; this abnormal expression may contribute to the development of arrhythmias. Second, glucocorticoids can activate the renin–angiotensin–aldosterone system (RASS) system, leading to increased blood pressure, rapid heart rate [62, 63]. High doses of glucocorticoids over-activate the RASS system and the sympathetic nervous system, increasing the risk of arrhythmias. Third, intravenous methylprednisolone alters myocardial cell stimulation thresholds and sodium metabolism. These changes may lead to the variation of electrolyte shifts in the membranes of the heart muscle, result in arrhythmias [64, 65]. Fourth, the use of high doses of glucocorticoids can induce insulin resistance, which may prevent glucose from entering cardiac muscle cells, aggravate an ischemic injury, and induce arrhythmias [49].Low-doses of glucocorticoids are less likely to cause these adverse reactions and may have a better control effect on POAF. We investigated the influence of glucocorticoids on AF after different types of surgery, glucocorticoids reduced the incidence of POAF in CABG group, but the effect of glucocorticoids in combined group was not statistically significant. We hypothesized that this may be due to the lower incidence of POAF and the lower inflammatory response in CABG alone.

The main side effects of glucocorticoids are gastrointestinal bleeding and an high risk of infection, which limits the application of glucocorticoids in heart surgery. We also conducted a meta-analysis of these risks, 12,126 cases were enrolled in 15 studies to assess the effects of glucocorticoids on infections including mediastinal, pulmonary, digestive and urinary tract infections, suggesting that glucocorticoids use did not lead to an higher risk of infection. Besides, a total of 11,155 patients were enrolled in 8 studies to assess the effect of glucocorticoids on gastrointestinal injury, including postoperative gastrointestinal bleeding, vomiting, gastrointestinal ulceration, or gastrointestinal dysfunction, meta-analysis results suggested that the effect of glucocorticoids on gastrointestinal injury was not significant. The present results seem to be at variance with our past consensus, and we speculate that this may be due to the shorter duration of glucocorticoid use in all studies and the smaller single dose, which needs to be confirmed in more and larger controlled trials. In addition to increased risk of infection and gastrointestinal damage, the use of glucocorticoids may cause abnormal glucose tolerance, electrolyte disorder, abnormal fat metabolism, osteoporosis, slow wound healing, nervous system abnormalities, and even lead to thromboembolism. When the drug is stopped for a long time, it can also lead to withdrawal reaction, adrenal cortical dysfunction, hypotension, and even shock. These side effects also limit the further use of glucocorticoids.

## Limitations

Firstly, the heterogeneity of the primary end points was too high. Subgroup analysis was conducted, but our grouping criteria were established in advance. In the second subgroup analysis, we found a significant reduction in heterogeneity in the CABG group, in the CABG OR Valvular Surgery group, the heterogeneity was significantly higher than that in the CABG group due to the inclusion of patients undergoing combined surgery. And in valve surgery, different valve lesions have different effects on cardiac function. Therefore, we believe that the differences in surgical methods are the main reason for the heterogeneity. In future trials, we plan to conduct more rigorous surgical classification to reduce heterogeneity. Due to the differences in drug sensitivity among the included population and the large differences in the dose of glucocorticoids used in each subgroup, this may be another factor leading to the generation of heterogeneity. We analyzed and discussed the source of heterogeneity in Limitations and finally choose to use random effect model to synthesize heterogeneity, and we cautiously recommend the final conclusion. Secondly, there was a lack of recent large clinical studies.

## Conclusions

The results of this study showed that glucocorticoids were beneficial in reducing the effect of POAF, and low-dose glucocorticoids could reduce the incidence of POAF. However, heterogeneity of overall study and subgroup analysis was high, so we held a cautious recommendation. Our study also did not find that glucocorticoids use increased the risk of infection and gastrointestinal injury.

## Supplementary Information

**Additional file 1.** Literature retrieval Method.

## Data Availability

We declare that the data supporting the conclusions of this article are fully described within the article.

## Abbreviations

POAF: Postoperative atrial fibrillation
CRP: C-reactive protein
CABG: Coronary artery bypass graft
RCT: Randomized controlled trial
PRISMA: The Preferred reporting items for systematic reviews and Meta-Analyses
CI: Confidence interval
RR: Relative risk
LTCC: L-type calcium Channel
SERCA2: Sarcoplasmic/endoplasmic reticulum calcium adenosine triphosphatase 2
NCX1: Sodium/calcium exchanger 1
RASS: Renin–angiotensin–aldosterone system
RoB 2: Cochrane risk-of-bias assessment tool (RoB 2)
