# Supplementary material for: Effects of different doses of glucocorticoids on postoperative atrial fibrillation: a meta-analysis
Source: BMC Cardiovasc Disord. 2023 Jan 12;23:16. doi: 10.1186/s12872-022-03001-0 (PMC9838011; doi:10.1186/s12872-022-03001-0)
Supplement: Supplementary file 1 — Additional file 1. Literature retrieval Method. [file 12872_2022_3001_MOESM1_ESM.docx]

We will search the following databases for the relevant English language literature：PubMed（MEDLINE），The Cochrane Central Register of Controlled Trials（CENTRAL）, Embase and Web Of Science.

- P：Postoperative atrial fibrillation or POAF or (cardiac surgery or Coronary Artery Bypass Grafting or CABG or cardiac valve surgery)
- I :Glucocorticoids or steroid or corticosteroid or dexamethasone or prednisolone or prednisone or methylprednisolone or hydrocortisone
- C:placebo
- S:(Randomized controlled trial or controlled clinical trial or randomized or placebo or clinical trials as topic or randomly or trial) not (animals not humans)

Retrieval strategy:(((("postoperative period"[MeSH Terms] OR ("postoperative"[All Fields] AND "period"[All Fields]) OR "postoperative period"[All Fields] OR "postop"[All Fields] OR "postoperative"[All Fields] OR "postoperatively"[All Fields] OR "postoperatives"[All Fields]) AND ("atrial fibrillation"[MeSH Terms] OR ("atrial"[All Fields] AND "fibrillation"[All Fields]) OR "atrial fibrillation"[All Fields])) OR "POAF"[All Fields] OR ("thoracic surgery"[MeSH Terms] OR ("thoracic"[All Fields] AND "surgery"[All Fields]) OR "thoracic surgery"[All Fields] OR ("cardiac"[All Fields] AND "surgery"[All Fields]) OR "cardiac surgery"[All Fields] OR "cardiac surgical procedures"[MeSH Terms] OR ("cardiac"[All Fields] AND "surgical"[All Fields] AND "procedures"[All Fields]) OR "cardiac surgical procedures"[All Fields] OR ("cardiac"[All Fields] AND "surgery"[All Fields]) OR ("coronary artery bypass"[MeSH Terms] OR ("coronary"[All Fields] AND "artery"[All Fields] AND "bypass"[All Fields]) OR "coronary artery bypass"[All Fields] OR ("coronary"[All Fields] AND "artery"[All Fields] AND "bypass"[All Fields] AND "grafting"[All Fields]) OR "coronary artery bypass grafting"[All Fields]) OR "CABG"[All Fields] OR (("heart valves"[MeSH Terms] OR ("heart"[All Fields] AND "valves"[All Fields]) OR "heart valves"[All Fields] OR ("cardiac"[All Fields] AND "valve"[All Fields]) OR "cardiac valve"[All Fields]) AND ("surgery"[MeSH Subheading] OR "surgery"[All Fields] OR "surgical procedures, operative"[MeSH Terms] OR ("surgical"[All Fields] AND "procedures"[All Fields] AND "operative"[All Fields]) OR "operative surgical procedures"[All Fields] OR "general surgery"[MeSH Terms] OR ("general"[All Fields] AND "surgery"[All Fields]) OR "general surgery"[All Fields] OR "surgery s"[All Fields] OR "surgerys"[All Fields] OR "surgeries"[All Fields])))) AND "clinical trial"[Publication Type] AND (("glucocorticoids"[Pharmacological Action] OR "glucocorticoids"[MeSH Terms] OR "glucocorticoids"[All Fields] OR "glucocorticoid"[All Fields] OR ("steroidal"[All Fields] OR "steroidals"[All Fields] OR "steroidic"[All Fields] OR "steroids"[MeSH Terms] OR "steroids"[All Fields] OR "steroid"[All Fields]) OR ("adrenal cortex hormones"[MeSH Terms] OR ("adrenal"[All Fields] AND "cortex"[All Fields] AND "hormones"[All Fields]) OR "adrenal cortex hormones"[All Fields] OR "corticosteroid"[All Fields] OR "corticosteroids"[All Fields] OR "corticosteroidal"[All Fields] OR "corticosteroide"[All Fields] OR "corticosteroides"[All Fields]) OR ("dexamethason"[All Fields] OR "dexamethasone"[MeSH Terms] OR "dexamethasone"[All Fields] OR "dexamethasone s"[All Fields] OR "dexamethasones"[All Fields]) OR ("prednisolon"[All Fields] OR "prednisolone"[MeSH Terms] OR "prednisolone"[All Fields]) OR ("prednison"[All Fields] OR "prednisone"[MeSH Terms] OR "prednisone"[All Fields]) OR ("methylprednisolone"[MeSH Terms] OR "methylprednisolone"[All Fields] OR "methylprednisolon"[All Fields]) OR ("hydrocortisone"[MeSH Terms] OR "hydrocortisone"[All Fields] OR "hydrocortisones"[All Fields])) AND "clinical trial"[Publication Type]) AND ((("randomized controlled trial"[Publication Type] OR "randomized controlled trials as topic"[MeSH Terms] OR "randomized controlled trial"[All Fields] OR "randomised controlled trial"[All Fields] OR ("controlled clinical trial"[Publication Type] OR "controlled clinical trials as topic"[MeSH Terms] OR "controlled clinical trial"[All Fields]) OR ("random allocation"[MeSH Terms] OR ("random"[All Fields] AND "allocation"[All Fields]) OR "random allocation"[All Fields] OR "random"[All Fields] OR "randomization"[All Fields] OR "randomized"[All Fields] OR "randomisation"[All Fields] OR "randomisations"[All Fields] OR "randomise"[All Fields] OR "randomised"[All Fields] OR "randomising"[All Fields] OR "randomizations"[All Fields] OR "randomize"[All Fields] OR "randomizes"[All Fields] OR "randomizing"[All Fields] OR "randomness"[All Fields] OR "randoms"[All Fields]) OR ("placeboes"[All Fields] OR "placebos"[MeSH Terms] OR "placebos"[All Fields] OR "placebo"[All Fields]) OR ("clinical trials as topic"[MeSH Terms] OR ("clinical"[All Fields] AND "trials"[All Fields] AND "topic"[All Fields]) OR "clinical trials as topic"[All Fields]) OR "randomly"[All Fields] OR ("clinical trials as topic"[MeSH Terms] OR ("clinical"[All Fields] AND "trials"[All Fields] AND "topic"[All Fields]) OR "clinical trials as topic"[All Fields] OR "trial"[All Fields] OR "trial s"[All Fields] OR "trialed"[All Fields] OR "trialing"[All Fields] OR "trials"[All Fields])) NOT (("animals"[MeSH Terms:noexp] OR "animals"[All Fields]) NOT ("human s"[All Fields] OR "humans"[MeSH Terms] OR "humans"[All Fields] OR "human"[All Fields]))) AND "clinical trial"[Publication Type])) AND (clinicaltrial[Filter])
